# Quantitative assessment of velocity and flow using compressed SENSE in children and young adults with adequate acquired temporal resolution

**DOI:** 10.1186/s12968-021-00811-7

**Published:** 2021-10-18

**Authors:** Murat Kocaoglu, Amol Pednekar, Jean A. Tkach, Michael D. Taylor

**Affiliations:** 1 Department of Radiology, Cincinnati Children’s Hospital Medical Center, S1.533, 3333 Burnet Ave, Cincinnati, OH 45229 USA; 2 Department of Radiology, University of Cincinnati College of Medicine, Cincinnati, OH USA; 3 The Heart Institute, Cincinnati Children’s Hospital Medical Center, Cincinnati, OH USA; 4 Department of Pediatrics, University of Cincinnati College of Medicine, Cincinnati, OH USA

**Keywords:** Compressed SENSE, Velocity and flow measurements, Temporal resolution, Pediatric, Children

## Abstract

**Background:**

Phase contrast (PC) cardiovascular magnetic resonance (CMR) imaging with parallel imaging acceleration is established and validated for measuring velocity and flow. However, additional acceleration to further shorten acquisition times would be beneficial in patients with complex vasculature who need multiple PC-CMR measurements, especially pediatric patients with higher heart rates.

**Methods:**

PC-CMR images acquired with compressed sensitivity encoding (C-SENSE) factors of 3 to 6 and standard of care PC-CMR with sensitivity encoding (SENSE) factor of 2 (S2) acquired as part of clinical CMR examinations performed between November 2020 and January 2021 were analyzed retrospectively. The velocity and flow through the ascending aorta (AAo), descending aorta (DAo), and superior vena cava (SVC) in a transverse plane at the level of pulmonary artery bifurcation were compared. Additionally, frequency power distribution and dynamic time warp distance were calculated for these acquisitions. To further validate the adequate temporal resolution requirement, patients with S2 PC-CMR in the same acquisition plane were added in frequency power distribution analysis.

**Results:**

Twenty-eight patients (25 males; 15.9 ± 1.9 years; body surface area (BSA) 1.7 ± 0.2 m^2^; heart rate 81 ± 16 bpm) underwent all five PC-CMR acquisitions during the study period. An additional 22 patients (16 males; 17.5 ± 7.7 years; BSA 1.6 ± 0.5 m^2^; heart rate 91 ± 16 bpm) were included for frequency power spectrum analysis. As expected, scan time decreased with increasing C-SENSE acceleration factor = 3 (37.5 ± 6.5 s, 26.4 ± 7.6%), 4 (28.1 ± 4.9 s, 44.7 ± 5.6%), 5 (21.6 ± 3.6 s, 57.6 ± 4.4%), and 6 (19.1 ± 3.2 s, 62.3 ± 4.2%) relative to SENSE = 2 (51.3 ± 10.1 s) PC-CMR acquisition. Mean peak velocity, net flow, and cardiac output were comparable (*p* > 0.87) between the five PC-CMR acquisitions with mean differences less than < 4%, < 2%, and < 3% respectively. All individual blood vessels showed a non-significant dependence of difference in *f*_max99_ (< 4 Hz, *p* > 0.2), and dynamic time warp distance (*p* > 0.3) on the C-SENSE acceleration factor used. There was a strongly correlated (r = 0.74) increase in *f*_max99_ (10.5 ± 2.2, range: 7.1–16.4 Hz) with increasing heart rate. The computed minimum required cardiac phase number was 15 ± 2.0 (range: 11–20) over the heart rate of 86 ± 15 bpm (range: 58–113 bpm).

**Conclusions:**

Stroke volume, cardiac output, and mean peak velocity measurements using PC-CMR with C-SENSE of up to 6 agree with measurements by standard of care PC-CMR with SENSE = 2 and resulted in up to a 65% reduction in acquisition time. Adequate temporal sampling can be ensured by acquiring 20 cardiac phases throughout the entire cardiac cycle over a wide range of pediatric and young adult heart rates.

## Introduction

Measuring cardiovascular flow is important, especially in pediatric population, for both congenital and acquired heart disease [1]. Time-resolved phase contrast (PC) cardiovascular magnetic resonance (CMR) imaging allows measurement of blood velocities, volume flow rate, total flow, and pressure gradients in the major cardiothoracic vasculature and heart. It is considered the clinical reference standard for quantification of blood flow [2–5]. Although accelerated PC-CMR scans can be performed in a breath-hold, inaccuracies can be introduced due to decreased temporal and spatial resolution [6, 7] and alteration in preload and afterload due to suspended respiration can affect the function and flow dynamics [8–12]. The inaccuracies and extent of flow alteration depends on breath-hold duration, heart rate, imaging parameters, and underlying comorbidities. PC-CMR acquisitions performed during normal breathing using either respiratory gating [13] or signal averaging [14] can help mitigate some of these physiologic effects. Alternatively, a free breathing retrospectively cardiac gated k-space segmented two-dimensional PC-CMR acquisition with a parallel imaging acceleration factor of 2 may be the preferred sequence for quantitative flow assessment over the cardiac cycle particularly in pediatric patients, many of whom find it difficult to sustain steady respiratory suspension of adequate duration.

Scan times of over a minute for each PC-CMR acquisition are typical, the exact duration is determined by the patient’s heart rate and acquired spatial and temporal resolution. Especially, the temporal resolution requirements of PC-CMR are more stringent in higher heart rates typically observed in pediatric population. Additionally, certain congenital heart diseases require PC-CMR measurement be made for multiple vessels, resulting in long exam durations [15, 16].

A number of acceleration techniques have been used to shorten PC-CMR scan times, including radial and spiral acquisition [17, 18], view sharing [19], parallel imaging with regular spatial [20–22] and spatio-temporal [23–25] undersampling, and sparse data undersampling with iterative reconstruction [26–28]. The effective temporal resolution of a PC-CMR acquisition is determined by the k-space sampling scheme reflecting the associated k-space weighted properties of the velocity mapping [19]. Notably, the effective temporal resolution determines the extent of low pass filtering of the velocity profile and hence the ability to accurately resolve the blood flow waveform and measure the peak velocity over the cardiac cycle [6, 7, 29]. In the normal heart, the time interval between the start of depolarization and the end of repolarization of the myocardium varies inversely with the heart rate [30, 31]. Thus, the frequency response, and hence waveform fidelity, of time resolved blood flow measurements using accelerated PC-CMR sequences need to be validated for specific acceleration approach over the wide range of clinically observed heart rates across pediatric and young adult population.

The primary purpose of our study was to test the hypothesis that compressed sensitivity encoding (C-SENSE) acceleration, employing a pseudorandom variable density undersampling of k-space in the spatial domain, provides accurate velocity and flow quantification, and maintains blood waveform fidelity using retrospectively cardiac gated PC-CMR imaging, in pediatric population. The secondary purpose was to investigate the adequate temporal resolution requirement for accurate measurement of peak velocity and flow over the wide range of heart rates observed in pediatric and young adult population.

## Materials and methods

This HIPAA-compliant, retrospective study was approved by the institutional review board (IRB) at our institution which waived informed consent. All the data and information were always under the control of our institution.

### Patients

We identified all pectus excavatum patients who had undergone clinically indicated CMR examinations that included PC-CMR with SENSE and with C-SENSE sequences between November 2020 and January 2021. During this period, C-SENSE was used as part of a clinical quality improvement project to shorten PC-CMR scan times. Only patients with structurally normal hearts and vasculature were included. To further validate the correlation between heart rate and maximum frequency content of aortic blood flow waveform with uniform sampling over a typical range of heart rate observed in pediatric and young adult patients, standard of care PC-CMR images of additional clinical patients obtained in the same acquisition plane were added in the frequency power distribution analysis component of the study. IRB approval was obtained for the current study which involved systematic retrospective review of images previously obtained for clinical quality improvement and standard clinical protocol.

### CMR technique

All CMR examinations were performed with a 1.5 T CMR scanner (Ingenia, Philips Healthcare, Best, The Netherlands). As part of the standard clinical protocol, breath-hold left ventricular (LV) short-axis (SAx) cine balanced steady state free precession (bSSFP) acquisitions covering the entire heart were performed using vector electrocardiogram gating with a dedicated 28 element torso coil and a respiratory bellows placed at the mediastinum. These breath-hold, retrospectively cardiac gated cine SAx acquisitions with prospective arrhythmia rejection were performed with a sensitivity encoding (SENSE) acceleration factor (R) of 2. The SAx cine imaging parameters were: repetition time (TR) ms/echo time (TE) ms, 2.5–2.7/1.25–1.35; flip angle (FA), 60°; acquired voxel size, 1.6–1.7 × 1.6–1.7 × 6–8 mm^3^ (zero gap); acquired temporal resolution, 37–45 ms. Free breathing retrospectively cardiac gated single-slice PC-CMR images were obtained in a transverse plane at the level of the pulmonary artery bifurcation, without any respiratory motion synchronization or compensation. A radiofrequency spoiled gradient recalled echo (GRE) sequence using a symmetric pair of 1–1 bipolar trapezoidal gradient waveforms for velocity encoding applied in the foot to head (i.e. slice) direction was used for data acquisition. Velocity (1st order) compensation was applied in the other two directions. The first PC-CMR scan was acquired using SENSE with R = 2 (S2) as the standard protocol; subsequent PC-CMR scans were acquired using C-SENSE with R = 3 (CS3), 4 (CS4), 5 (CS5), and 6 (CS6), respectively. The common PC-CMR imaging parameters were: TR/TE—4.2/2.5; FA, 12°; turbo factor = 3–4, acquired voxel size, 1.8–2 × 1.8–2 × 8 mm^3^; reconstructed voxel size, 1.25 × 1.25–2 × 8 mm^3^; acquired temporal resolution, 25.2–33.6 ms; reconstructed temporal resolution, 25 ms (22–30 cardiac phases); velocity encoding, 150 cm/s. Thus, the point of 50% degradation in the frequency response of the blood velocity signal, is *f*_3dB_ = 13–17.5 Hz [6]. All cine bSSFP and PC-CMR imaging was performed without contrast. Acquisition duration and average heart rate were extracted from the scanner log files.

The commercially available implementation of SENSE and C-SENSE were used for the PC-CMR acquisitions and real-time reconstruction on the scanner console. Specifically, for the PC-CMR sequence, k-space is traversed in a linear sequence of phase encoding segments. SENSE uses a regular ky undersampling pattern along with coil sensitivity information, and spatial solution space constraint based on prior knowledge of the image extent. Whereas C-SENSE uses an irregular ky undersampling pattern determined by a pseudo-random variable density Poisson disc distribution for the prescribed field of view and spatial resolution along with the SENSE reconstruction algorithm using iterative reconstruction and sparsity constraints [32, 33]. In both acceleration techniques, predetermined k_y_ lines are then divided into small sequential k-space segments of 3–4 k_y_ lines each, based on the prescribed turbo factor. Thus, with C-SENSE acceleration, central k-space is almost fully sampled and the phase encoding gradient amplitude change is minimal close to the center of k-space, and phase inaccuracies due to eddy currents are confined to the periphery of k-space. In both acceleration techniques, each k-line was acquired with interleaved inverted polarity of the bipolar velocity encoding gradients. The k-space segment is repeated for each cardiac phase and incremented after each cardiac cycle with prospective filtering by prescribed arrhythmia rejection criteria. For the patient PC-CMR acquisitions evaluated for this study, the entire set of predetermined k_y_ lines was acquired sequentially 3 times. Each individual cardiac cycle was resampled linearly over the average cardiac cycle and signal averaging was performed across the 3 successive acquisitions prior to image reconstruction. Thus, physiologic variations in blood flow over 20 to 25 heart beats were averaged. Complex subtraction of the images acquired with interleaved symmetric velocity sensitivities was performed to generate phase contrast images. Spatially dependent phase error due to concomitant fields and residual eddy currents was corrected using concomitant field correction [34] followed by local phase correction filters [35].

### Image analysis

Standard image analysis was performed with a dedicated commercial system (cvi42, Circle Cardiovascular Imaging, Calgary, Alberta, Canada). The SAx cine and PC-CMR images were analyzed by a single reviewer (MK) with > 2 years of CMR and CMR post-processing experience. Quantitative assessment of LV stroke volume (SV) and cardiac output (CO) was performed on SAx cine images as detailed in [33]. For the PC images, smooth elastic contours were drawn manually on the end-diastolic phase of PC-CMR images to delineate ascending aorta (AAo), descending aorta (DAo), and superior vena cava (SVC). These contours were then propagated across all cardiac phases. Where required, manual corrections were applied to correct for in-plane displacement and diameter changes. Mean blood velocity at each cardiac phase was calculated by averaging the phase signal over the vessel cross-sectional area. Volume flow was calculated by integrating the product of area and mean velocity within the contoured blood vessel. Individual blood flow velocity waveforms were exported for further analysis.

### Data analysis

Descriptive statistics of continuous quantitative measurements are summarized as means and standard deviations. Individual blood flow waveforms were normalized by the peak mean velocity in the corresponding S2 waveform. To accommodate for heart rate changes between the scans, waveform similarity was quantified as a distance metric using a dynamic time warping algorithm [36] between the S2 and corresponding CS3-6 waveforms. The frequency power spectrum analysis was performed on mean-detrended blood velocity waveforms [29]. The frequency below which 99% of the total signal energy of the waveform is contained was considered as the highest spectral content, indicated as *f*_max99_. Bland–Altman analysis [37] was used to compare LV stroke volume and CO obtained using planimetry against those obtained using five PC-CMR acquisitions, and to compare mean peak velocity and *f*_max99_ obtained using standard of care S2 PC-CMR against those computed using CS3, CS4. CS5, and CS6. Tukey box plots [38] were used to compare the differences in mean peak velocity, net flow, *f*_max99_, and dynamic time warp distance between S2 PC-CMR and the four C-SENSE PC-CMR acquisitions. To study the significance of differences in parameter values across C-SENSE acceleration factors and individual blood vessels, a linear mixed effects model was applied using values obtained with S2 PC-CMR as reference. C-SENSE acceleration factors and individual blood vessels were used as fixed effects and patients were used as random effects. Analysis of variance (ANOVA) testing was used to compare mean peak velocity, net flow, and computed *f*_max99_ across C-SENSE acceleration factors in the three blood vessels evaluated. Pearson’s linear correlation coefficient *r* between heart rate and AAo ejection period, *f*_max99_ were calculated. A *p*-value < 0.05 was considered significant for all inference testing and 95% confidence intervals were calculated as appropriate. Correlation coefficients were interpreted as follows: 0–0.19, very weak; 0.2–0.39, weak; 0.40–0.59, moderate; 0.60–0.79, strong; and 0.80–1.0, very strong [39]. All statistical and blood flow waveform analyses were performed using MATLAB (The MathWorks™ Inc., Natick, Massachusetts, USA).

## Results

Twenty-eight patients (25 males; 15.9 ± 1.9 year (range: 12–20 years), body surface area (BSA) 1.7 ± 0.2 (range: 1.3–2.0 m^2^), heart rate 81 ± 16 bpm (range: 58–111 bpm)) with structurally normal heart and vasculature underwent all five PC-CMR acquisitions during the study period. Twenty-two patients (16 males; 17.5 ± 7.7 year (range: 2–37 years); BSA 1.6 ± 0.5 (range: 0.5–2.7 m^2^); heart rate 91 ± 16 bpm (range: 66–113 bpm)) with standard of care PC-CMR with SENSE R = 2 were added in the frequency power spectrum analysis. Table 1 summarizes the patient characteristics.

**Table 1:** Characteristics of the study population

PC-CMR acquisitions	SENSE and C-SENSE	SENSE
Number of patients	28	22
Age (year)	15.9 ± 1.9 (12–20)	17.5 ± 7.7 (2–37)
Female-to-male ratio	3:25	6:16
Height (cm)	172.3 ± 11.9 (148–191)	154.4 ± 24.8 (77–194)
Weight (kg)	58.1 ± 12.0 (38–82)	62.8 ± 30.5 (9–157)
BSA (m^2^)	1.7 ± 0.2 (1.25–2.02)	1.6 ± 0.5 (0.45–2.74)
Heart rate (bpm)	81 ± 16 (58–111)	91 ± 16 (66–113)
Clinical indications		
Pectus excavatum	28	0
Duchenne muscular dystrophy	0	8
Dilated cardiomyopathy	0	2
Pericarditis	0	2
Aortapathy	0	2
Valvular heart disease	0	3
Coronary disease	0	2
Other	0	3

The data are presented as the mean ± standard deviation (minimum – maximum) or as the number of subjects. *bpm* beats/min, *BSA* body surface area, *R* acceleration factor

**Fig. 1:**
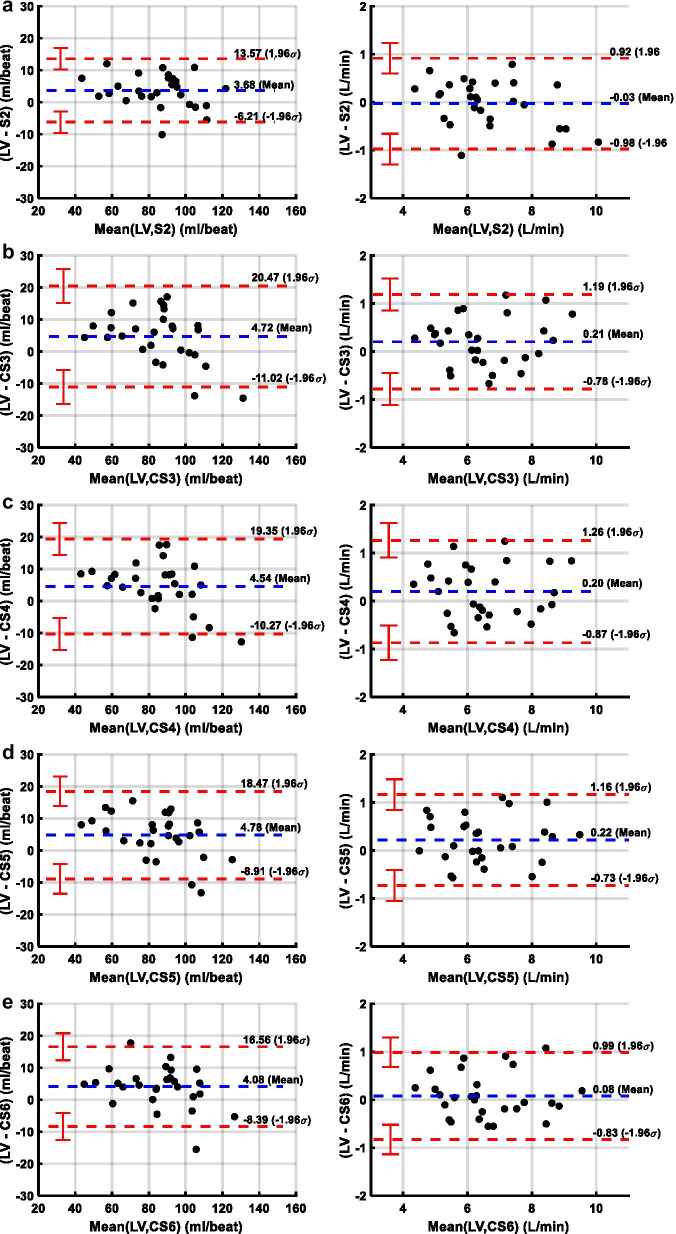
Bland–Altman plots comparing left ventricular stroke volume and cardiac output measured by planimetry using short axis stack of cine bSSFP images with that measured from PC-CMR images acquired with **a** SENSE = 2, **b** C-SENSE = 3, **c** C-SENSE = 4, **d** C-SENSE = 5, and **e** C-SENSE = 6. *CS3,4,5,6*  C-SENSE acceleration factor of 3,4,5,6, *LV*  left ventricle, *S2*  SENSE acceleration factor of 2

**Fig. 2:**
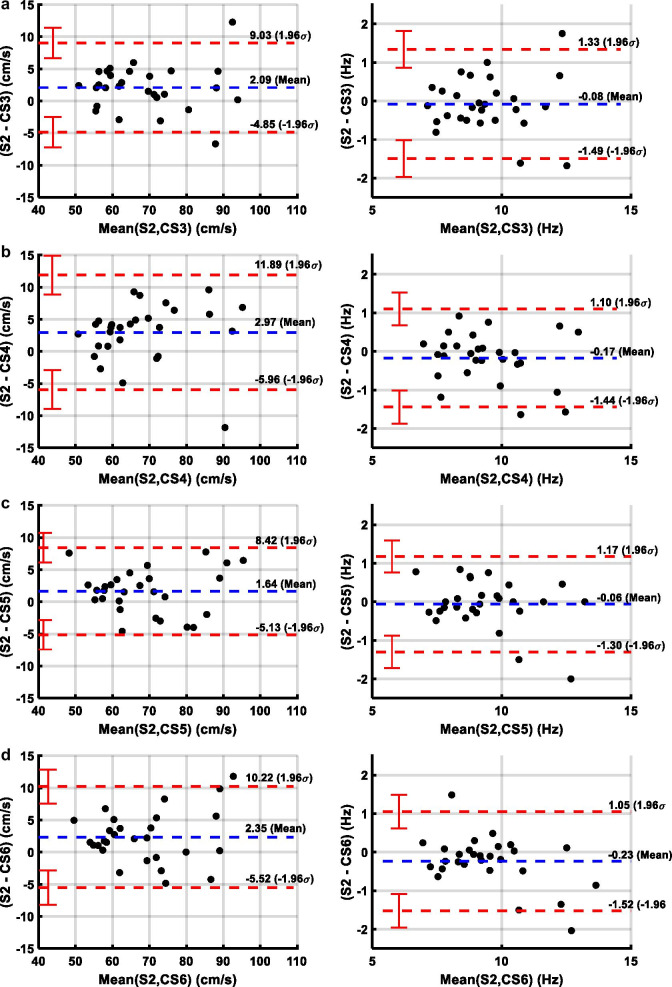
Bland–Altman plots comparing mean peak velocity and *f*_max99_ of ascending aorta obtained from SENSE = 2 PC-CMR compared to those obtained by (**a**) C-SENSE = 3, (**b**) C-SENSE = 4, (**c**) C-SENSE = 5, and (**d**) C-SENSE = 6. CS3,4,5,6 = C-SENSE acceleration factor of 3,4,5,6, *f*_max99_ = frequency below which 99% of the total signal energy of the waveform is contained, S2 = SENSE acceleration factor of 2

Table 2 demonstrates the data comparing heart rate, acquisition duration, AAo mean peak velocity, AAo net flow, and planimetry based cardiac output for the five PC-CMR and SAx cine imaging acquisitions. As expected, scan time decreased with increasing acceleration factor of C-SENSE = 3 (37.5 ± 6.5 s, 26.4 ± 7.6%), 4 (28.1 ± 4.9 s, 44.7 ± 5.6%), 5 (21.6 ± 3.6 s, 57.6 ± 4.4%), and 6 (19.1 ± 3.2 s, 62.3 ± 4.2%) with respect to SENSE = 2 (51.3 ± 10.1 s) PC-CMR acquisition. The absolute mean difference of all five PC-CMR acquisitions with respect to planimetry based measurements were less than 0.25 L/min (< 3%) for cardiac output and less than 5 ml/beat (< 6.5%) for LV stroke volume. Figure 1 depicts Bland–Altman plots comparing LV stroke volume and cardiac output computed by planimetry using SAx cine images with corresponding net AAo flow and cardiac output values obtained using the five PC-CMR acquisitions. The limits of agreement of all five PC-CMR acquisitions were less than 16 ml/beat (< 17.5%) for stroke volume and less than 1.07 L/min (< 16.5%) for cardiac output. Figure 2 depicts Bland–Altman plots comparing mean peak velocity and *f*_max99_ of AAo obtained using four C-SENSE PC-CMR acquisitions compared to SENSE = 2 PC-CMR. Mean peak velocity in AAo showed mean difference of less than 3 cm/s (< 4%) and limits of agreement less than 9 cm/s (< 12%) for all four C-SENSE PC-CMR acquisitions compared to SENSE = 2 PC-CMR. Computed *f*_max99_ in AAo had absolute mean difference of less than 0.25 Hz and limits of agreement less than 1.5 Hz for all four C-SENSE PC-CMR acquisitions compared to SENSE = 2 PC-CMR.

**Table 2:** Velocity and flow measurements and difference in the values between PC-CMR acquisitions in ascending aorta with SENSE acceleration factor of 2 and with C-SENSE acceleration factors of 3,4,5,6

	Heart rate (bpm)	Acquisition duration (s)	Mean peak velocity (cm/s)	Net flow or SV (ml/beat)	Cardiac output (L/min)
LV	78 ± 14			87.5 ± 18.6	6.7 ± 1.4
S2	82 ± 16	51.3 ± 10.1	69.1 ± 13.3	83.8 ± 19.8	6.7 ± 1.5
CS3	80 ± 16	37.5 ± 6.5	67.5 ± 12.7	82.8 ± 22.2	6.4 ± 1.3
CS4	80 ± 16	28.1 ± 4.9	66.6 ± 12.4	83.0 ± 22.2	6.5 ± 1.3
CS5	81 ± 16	21.6 ± 3.6	67.9 ± 12.6	82.7 ± 21.9	6.4 ± 1.3
CS6	81 ± 15	19.1 ± 3.2	67.3 ± 12.1	83.4 ± 20.6	6.6 ± 1.4
LV – S2	− 4.1 ± 5.3*			3.7 ± 5.1*	− 0.03 ± 0.48
LV – CS3 or S2 – CS3	− 3.0 ± 5.9*	13.9 ± 6.1*	2.1 ± 3.5*	4.7 ± 8.0*	0.21 ± 0.50*
LV – CS4 or S2 – CS4	− 2.9 ± 5.4*	23.2 ± 6.7*	3.0 ± 4.6*	4.5 ± 7.6*	0.20 ± 0.54*
LV – CS5 or S2 – CS5	− 3.1 ± 6.4*	29.8 ± 7.4*	1.6 ± 3.5*	4.8 ± 7.0*	0.22 ± 0.48*
LV – CS6 or S2 – CS6	− 3.5 ± 5.3*	32.2 ± 7.8*	2.4 ± 4.0*	4.1 ± 6.4*	0.08 ± 0.46

Unless otherwise indicated, data are means ± standard deviations. *CS3,4,5,6*  C-SENSE acceleration factor of 3,4,5,6, *LV*  left ventricle, *S2*  SENSE acceleration factor of 2, *SV*  stroke volume. The *represents *p* < 0.05

**Fig. 3:**
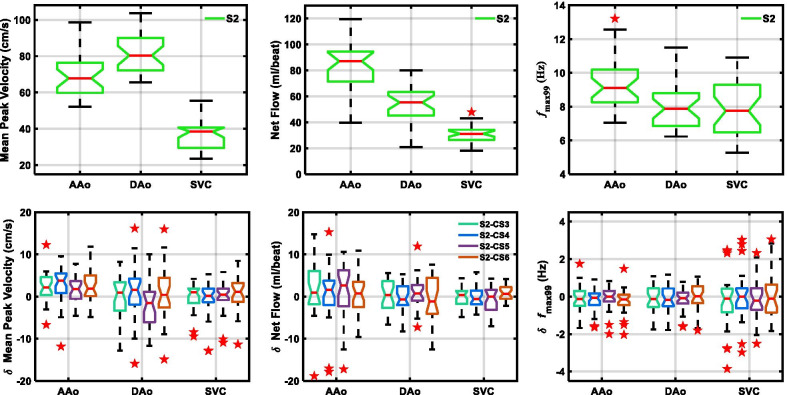
Tukey box plots of mean peak velocity (left), net flow (center), and *f*_max99_ (right) values obtained using SENSE = 2 PC-CMR (top row) and differences in those values obtained using four C-SENSE PC-CMR acquisitions (bottom row) for the three vessels evaluated. Horizontal red line = median, whiskers = minimum and maximum within 1.5 times interquartile distance, red star = outliers beyond 1.5 times the interquartile distance. For absolute values (top row), non-overlapping notches indicate that the medians of the two groups differ at the 5% significance level. For difference in values (bottom row), notches excluding zero indicate that there is non-zero bias at 5% significance level between two measurements. *δ*  difference in values, *AAo*  ascending aorta, *CS3, CS4, CS5, CS6*  C-SENSE acceleration factor of 3,4,5,6, *DAo*  descending aorta, *f*_max99_  frequency below which 99% of the total signal energy of the waveform is contained, *LV*  left ventricle, *S2*  SENSE acceleration factor of 2, *SVC*  superior vena cava

Distributions of mean peak velocity, net flow, and computed *f*_max99_ obtained by SENSE = 2 PC-CMR for AAo, DAo, and SVC and corresponding Tukey box plots comparing differences in those values obtained using four C-SENSE accelerated PC-CMR acquisitions with respect to SENSE = 2 PC-CMR are depicted in Fig. 3. Differences in these values across C-SENSE acceleration factors in all three individual blood vessels were non-significant (*p* > 0.07), except for mean peak velocity in AAo (*p* < 0.05).

**Fig. 4:**
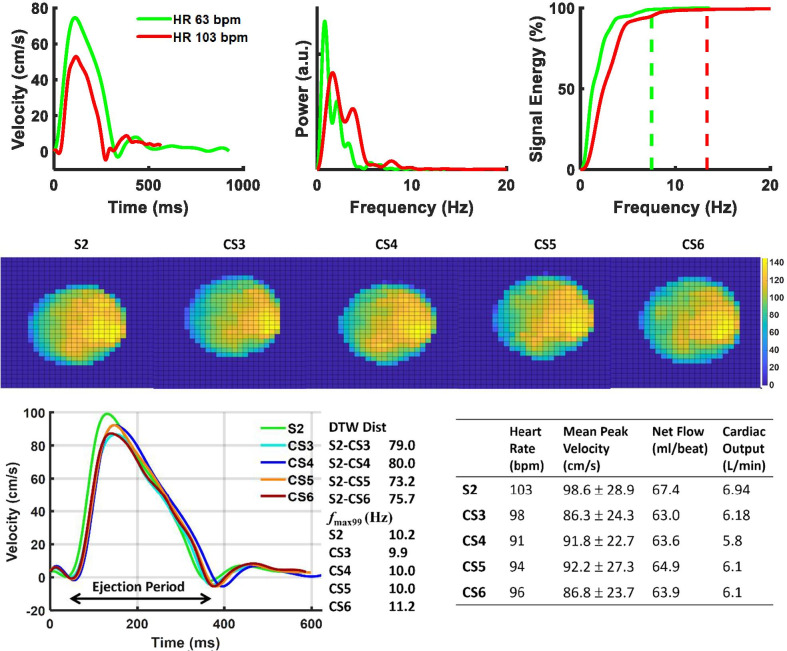
Representative blood flow velocity waveforms in ascending aorta with corresponding power spectra, cross-sectional velocity maps, and dynamic time warp distance. Top Row: Blood velocity waveforms (left) with S2 PC-CMR from patients with heart rate of 63 bpm (10th percentile) and 103 bpm (90th percentile), corresponding power spectra (center), and cumulative sum of power spectra (right) with the dotted vertical lines indicating the frequency below which 99% of the total signal energy of the waveform is contained, 7.5 Hz and 13.3 Hz respectively. Middle row: Pixel-wise velocity maps in ascending aorta for patient number 20 with different acceleration factors. Bottom row: mean blood flow velocity waveforms (left) associated with the middle row, dynamic time warp distance and *f*_max99_ (center), and heart rate, mean peak velocity, net flow, and cardiac output (right) for each PC-CMR scan. CS3,4,5,6 = C-SENSE acceleration factor of 3,4,5,6, DTW Dist = dynamic time warp distance, *f*_max99_ = frequency below which 99% of the total signal energy of the waveform is contained, S2 = SENSE acceleration factor of 2, Ejection period = foot-to-foot distance in ascending aortic blood waveform

**Fig. 5:**
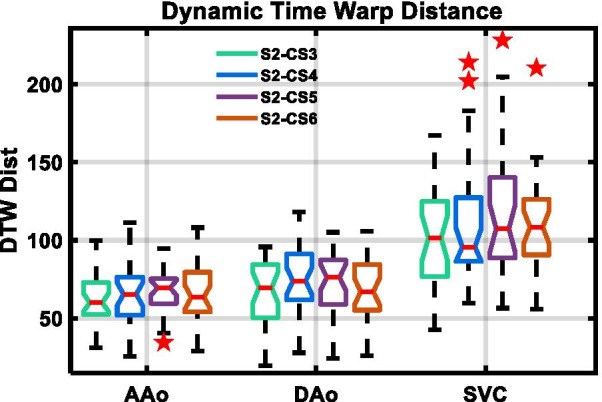
Tukey box plots dynamic time warp distance for four C-SENSE PC-CMR acquisitions with respect to S2 PC-CMR. Horizontal red line = median, whiskers = minimum and maximum within 1.5 times interquartile distance, red star = outliers beyond 1.5 times the interquartile distance. Non-overlapping notches indicate that the medians of the two groups differ at the 5% significance level. *AAo*  ascending aorta, *CS3,4,5,6*  C-SENSE acceleration factor of 3,4,5,6, *DAo*  descending aorta, *S2*  SENSE acceleration factor of 2, *S2-CS3,4,5,6*  comparison CS3,4,5,6 with respect to S2, *SVC*  superior vena cava

Figure 4 depicts representative blood flow velocity waveforms in the AAo with corresponding power spectra, cross-sectional velocity maps, and dynamic time warp distances. The top row demonstrates the shorter ejection period (340 ms, 290 ms), defined as foot-to-foot distance of the AAo blood waveform, and increased *f*_max99_ (7.5 Hz, 13.3 Hz) observed with increased heart rates (63 bpm, 103 bpm). In the middle row, slight spatial blurring is observed in the C-SENSE PC-CMR velocity maps following the application of concomitant field correction and local phase correction filters. Physiologic variations and quantitative measurements over the corresponding PC-CMR scans are depicted in the bottom row. Tukey box plots comparing dynamic time warp distance for all patients and individual vessels obtained using four C-SENSE accelerated PC-CMR acquisitions with respect to SENSE = 2 PC-CMR are depicted in Fig. 5. The linear mixed effects model resulted in negligible variance explained by the random effects (patients). Although, the dynamic time warp distance varied significantly (*p* < 0.0001) across individual blood vessels, the effect of C-SENSE acceleration factor within individual blood vessel was non-significant (*p* > 0.4).

**Fig. 6:**
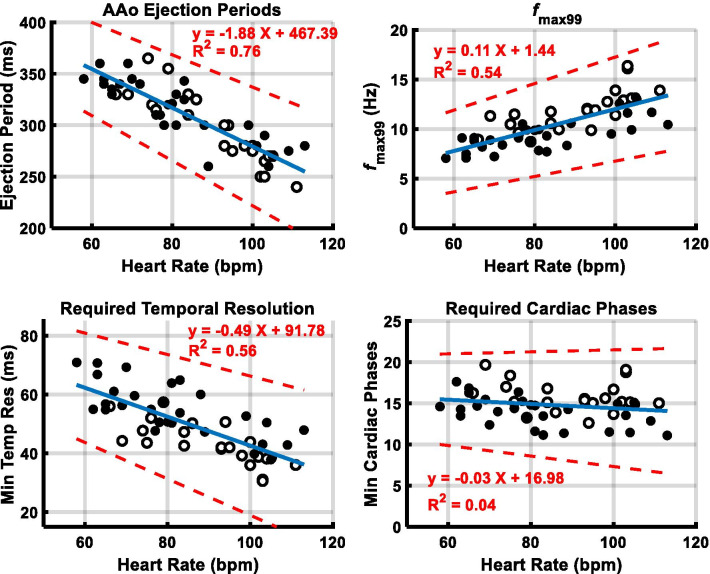
Plots of measured AAo ejection period, and computed *f*_max99,_ minimum required temporal resolution, and minimum required cardiac phases in S2 PC-CMR acquisitions against heart rate. Black circle = patients with structurally normal heart, open circle = additional patients with various clinical indications, AAo = ascending aorta, Ejection period = foot-to-foot distance of AAo blood waveform, *f*_max99_ = frequency below which 99% of the total signal energy of the waveform is contained, S2 = SENSE acceleration factor of 2

Figure 6 shows the measured AAo ejection period, computed *f*_max99,_ minimum required temporal resolution and minimum required cardiac phases in S2 PC-CMR acquisitions versus heart rate in 28 structurally normal patients, and additional 22 patients with various clinical indications. Between these two cohorts, there was no significant difference in Pearson’s linear correlation coefficients between heart rate and ejection period (*p* > 0.6), *f*_max99_ (*p* > 0.9), minimum required temporal resolution (*p* > 0.8), or minimum required cardiac phases (*p* > 0.2). For the two cohorts pooled together, there was strong to very strong correlations between heart rate and ejection period (*r* = − 0.87), *f*_max99_ (*r* = 0.74), and minimum required temporal resolution (*r* = − 0.75). There was an inverse relationship between ejection period (307 ± 33, range: 240–365 ms) and heart rate with a corresponding direct relationship of *f*_max99_ (10.5 ± 2.2, range: 7.1–16.4 Hz) with heart rate. Thus, the computed minimum required temporal resolution (50 ± 10, range: 31–71 ms) increased with heart rate. The corresponding computed minimum required cardiac phase number was 15 ± 2 (range: 11–20) over the heart rate 86 ± 15 bpm (range: 58–113 bpm).

## Discussion

There were two major findings of this compressed-SENSE accelerated phase contrast imaging and frequency spectrum analysis study. First, velocity and volume flow rate measurements over a wide range of heart rates in the major cardiothoracic blood vessels using PC-CMR with C-SENSE acceleration factors up to 6 provided values comparable to those obtained with the standard of care acquisition using a SENSE acceleration factor of 2 with up to 65% reduction in scan time. Second, adequate temporal sampling with preserved velocity waveforms can be ensured by acquiring 20 cardiac phases throughout the entire cardiac cycle over the wide range of clinically observed heart rates across pediatric and young adult population.

Total imaging time for PC-CMR can be quite long—on the order of one minute per interrogated blood vessel. This is especially burdensome when imaging complex cardiothoracic vasculature that require flow measurements in multiple vessels [15, 16]. Although, various data undersampling techniques can be employed to accelerate PC-CMR, the effective temporal resolution of the blood flow waveform measured by each technique will be governed by the specific k-space and k-t space sampling scheme reflecting the associated k-space weighted properties of the velocity mapping. Velocity induced phase shifts in the phase contrast technique are mainly encoded in the central one-eighth of k-space [19]. The central k-space velocities dominate the encoded velocities in the reconstructed phase images. Hence, optimizing the sampling rate of the central k-space region is critical for accurate measurement of the velocity in pulsatile waveform. Thus, the effects of data undersampling schemes on velocity and volume flow rate measurements need to be verified comprehensively. Since the effective temporal resolution of the central k-space region predominantly determines the frequency response, frequency spectrum analysis provides a method to systematically quantify the effects of acquisition scheme on the measurement of rapidly changing velocity and volume flow rates during systole [6, 7]. This is particularly important during the rapid ejection phase in the ascending aorta.

PC-CMR is well suited for acceleration using compressed sensing techniques. Most of the FOV is comprised of static tissue, and the temporal signal change is limited to regions of blood flow, resulting in sparse data following the application of the appropriate transform. Accurate velocity measurements with compressed sensing sampling a 30% of k-space data have been reported [40]. Since C-SENSE applies undersampling in the spatial domain only, via a pseudorandom variable density Poisson disc distribution, the central one-eighth of k-space is almost fully sampled for each time frame, even at high acceleration factors. The sampling pattern and percentage of k-space acquired in total is exclusively dependent on the C-SENSE acceleration factor for the prescribed field of view and spatial resolution and is completely independent of the turbo factor, aka number of k-lines collected per segment. As such, the turbo factor affects only the temporal fidelity of the measured blood flow waveform and acquisition duration.

In this study, k-space segments comprised of 3–4 k-lines for interleaved velocity encoding provided an acquired temporal resolution of 25–34 ms corresponding to 50% degradation in frequency response at *f*_3dB_ = 13–17.5 Hz [6]. Serial signal averaging separated individual acquisitions by ~ 20 heart beats. Thus, physiologic variations within each individual PC-CMR scan were averaged. Heart rates for the patients in this study ranged from 58 to 111 bpm; the corresponding ejection periods in the ascending aorta ranged from 340 to 290 ms. Frequency response and waveform similarity analysis in all individual blood vessels showed non-significant dependence of difference in *f*_max99_ (< 4 Hz, *p* > 0.2), and dynamic time warp distance (*p* > 0.3) on C-SENSE acceleration factor used, indicating preserved waveform fidelity across C-SENSE acceleration factors in all three individual blood vessels. Correspondingly, differences in mean peak velocity (*p* > 0.9) and net flow (*p* > 0.9) in all three blood vessels were independent of C-SENSE acceleration factor used. The underestimation of AAo mean peak velocity by all four C-SENSE PC-CMR acquisitions was less than 3 cm/s (< 4%) compared to S2 PC-CMR. The overestimation of LV stroke volume by SAx measurements compared to aortic flow is less than 5 ml/beat (< 2%), similar to previously reported values [33]. Cardiac output measured with PC-CMR correlated strongly with that measured using LV SAx cine imaging with less than 0.25 L/min (< 3%) mean difference. Some of these differences in mean peak velocity and volume flow rate can be attributed to physiologic variation corresponding to changes in heart rate from breath-hold LV SAx cine imaging compared with consecutive free breathing PC-CMR sequences in individual patients. The combination of exclusion of the coronary blood flow in the phase-contrast aortic measurement and the inclusion of papillary muscles in the LV volumetric calculation also contributes to these differences [41, 42]. Previous studies using phase contrast measurement in cardiopulmonary arteries suggested that a 3.5–5% deviation from the true flow, which is less than 4 mL in a patient with stroke volume of 80 mL is acceptable [43–46]. Overall, velocity and volume flow rate measurement across a wide range of heart rates in both fast and slow flowing major cardiothoracic vessels with C-SENSE 3, 4, 5, and 6 is feasible with clinically acceptable accuracy. Scan time decreased non-linearly with increasing C-SENSE acceleration factor, as expected. The PC-CMR scan duration was about 60 heart beats; the wide range of heart rates in our study population resulted in a relatively wide range of scan durations. Overall, incremental reduction in scan time between increasing consecutive C-SENSE acceleration factors greater than 4 were less than 10 s.

Our *f*_max99_ results for the ascending aorta for heart rates less than 70 bpm are in agreement with those reported in a recent study in adults [29]. We observed that reduction in ejection period, defined as foot-to-foot distance of the AAo blood waveform, correlated very strongly (*r* = − 0.87) with increasing heart rate. Frequency response analysis showed a strongly correlated (r = 0.74) increase in *f*_max99_ with increasing heart rate, indicating higher rate of change of aortic flow during the rapid ejection phase. Thus, the adequate required temporal resolution is dependent on the heart rate, seen in the range of patient heart rates observed in pediatric and young adult population. The computed minimum required cardiac phase number was 15 ± 2 (range: 11–20) over the heart rate range 86 ± 15 bpm (range: 58–113 bpm). This analysis suggests that for velocity and volume flow rate measurement in the aorta, adequate temporal sampling can be ensured by acquiring 20 cardiac phases over a range of heart rates observed in pediatric and young adult population.

This study was primarily designed to evaluate the effects of increasing C-SENSE acceleration factors on PC-CMR based flow measurements within the limited additional scan time available during routine CMR patient exams. Notably, additional factors such as velocity encoding value with respect to actual peak velocity, eddy current variation due to gradient strength, and slice orientation may influence the accuracy of flow measurements. Each of these factors will be explored systematically in future studies in which analogous standard and C-SENSE PC-CMR data will be collected in the presence of abnormal flow patterns and in double oblique planes. This study has several limitations. First, our patient population was a cohort of pectus excavatum patients who did not have any structural cardiovascular or valvular abnormalities, and all had normal flow profiles. In patients with stenoses or regurgitation, flow profiles can change due to multidirectional flow secondary to signal saturation and dephasing caused by inhomogeneous in-plane and/or recirculating flow [47]. Second, the PC-CMR with different C-SENSE acceleration factors were acquired with an identical velocity encoding value and only in the transverse plane in all patients. For a given patient, one would expect that the differences in flow measurements due to differences in eddy current effects between SENSE and C-SENSE acquisitions to remain similarly negligible across orientations of acquisition plane as the concomitant gradient terms should remain the same between the two PC-CMR acquisitions. The increased signal available with contrast agents and/or higher field strengths may help reduce spatial blurring at higher C-SENSE acceleration factors—particularly beneficial for smaller caliber blood vessels. Third, we did not have external flow data from any other modality such as Doppler ultrasound, or PC-CMR data with fully sampled k-space. However, stroke volume and cardiac output measured from breath-hold LV SAx were used as an internal reference to partially address this limitation. Fourth, breath-hold LV SA acquisitions were followed by consecutive PC-CMR scans with increasing acceleration factors. Thus, the effect of respiratory suspension on cardiac sinus rhythm and subsequent return to a free breathing heart rate may have introduced a systematic change in stroke volume across the scans. The acquisition of a free breathing short-axis cine stack could have mitigated the physiologic effects associated with breath-holds. However, it is likely that the differences in measured stroke volume and net aortic flow between breath-hold and free breathing are small relative to differences introduced by the uncertainties in ventricular contouring, inclusion of papillary muscles and trabeculations in the blood volume, and the contribution of coronary artery flow [41, 42]. In clinical practice, we use breath-hold cine acquisition as the default method and free breathing cine acquisition [33] when breath-holding is not feasible. We accept the possible difference in stroke volumes between breath-hold and free-breathing in favor of fewer artifacts and better overall image quality with breath-holds compared to free breathing [33]. Also, the free breathing cine sequence requires activation/ deactivation, of a non-FDA approved CMR system software. Fifth, the acquired temporal resolution would ideally have been identical for all the patients. This however was not practical as it would have substantially prolonged scan durations in patients with lower heart rates.

## Conclusion

Stroke volume, cardiac output, and mean peak velocity measurements over a wide range of pediatric and young adult heart rates using retrospectively cardiac gated PC-CMR with a C-SENSE acceleration factor of up to 6 were comparable to those measured with cine imaging based LV volumetry and PC-CMR with SENSE acceleration factor of 2 and demonstrated up to a 65% reduction in acquisition time. Correspondingly, blood flow waveform fidelity and frequency spectra content for the major cardiothoracic blood vessels were preserved by C-SENSE acceleration. For both velocity and volume flow rate measurements in major cardiothoracic blood vessels, adequate temporal sampling can be ensured by acquiring 20 cardiac phases across the cardiac cycle. Encouraged by the results of the present study, additional clinical validation studies in larger cohorts of patients that include the evaluation of multiple blood vessels encompassing a wide range of peak velocities and complex flow patterns were initiated and are currently underway.

## Data Availability

The datasets generated and/or analyzed during the current study are not publicly available due to patient privacy concern and institutional policies but are available from the corresponding author on reasonable request.

## Abbreviations

AAo: Ascending aorta
ANOVA: Analysis of variance
BSA: Body surface area
bSSFP: Balanced steady state free precession
CS3,4,5,6: Compressed sensitivity encoding with R = 3,4,5,6
C-SENSE: Compressed sensitivity encoding
CO: Cardiac output
DAo: Descending aorta
*f*_max99_: Frequency below which 99% of the total signal energy of the waveform is contained
FA: Flip angle
GRE: Gradient recalled echo
LV: Left ventricle/left ventricular
PC-CMR: Phase contrast cardiovascular magnetic resonance
R: Acceleration factor
S2: Sensitivity encoding with R = 2
SAx: Short-axis
SENSE: Sensitivity encoding
SV: Stroke volume
SVC: Superior vena cava
TE: Echo time
TR: Repetition time
